# IKZF1基因缺失在急性B淋巴细胞白血病患者中的预后意义

**DOI:** 10.3760/cma.j.issn.0253-2727.2022.03.009

**Published:** 2022-03

**Authors:** 冰清 唐, 梓红 蔡, 岱楠 林, 治香 王, 晓杰 梁, 志平 范, 芬 黄, 启发 刘, 红升 周

**Affiliations:** 1 南方医科大学南方医院血液科，广州 510515 Department of Hematology, Nanfang Hospital, Southern Medical University, Guangzhou 510515, China; 2 广州市第十二人民医院血液科，广州 510515 Department of Hematology, Guangzhou Twelfth People's Hospital, Guangzhou 510515, China

**Keywords:** 基因，IKZF1, 白血病，B淋巴细胞，急性, 异基因造血干细胞移植, Gene, IKZF1, Leukemia, B-cell, acute, Allogeneic hematopoietic stem cell transplantation

## Abstract

**目的:**

分析IKZF1基因缺失在急性B淋巴细胞白血病（B-ALL）患者中的预后意义。

**方法:**

回顾性分析2016年3月至2019年9月南方医科大学附属南方医院收治的142例接受儿童样化疗方案的B-ALL患者的临床资料，分析IKZF1缺失患者的临床特征。无事件生存（EFS）和总生存（OS）的多因素分析使用Cox回归模型。将患者分为IKZF1缺失/单纯化疗组（A组）、IKZF1缺失/移植组（B组）、IKZF1非缺失/单纯化疗（C组）、IKZF1非缺失/移植（D组）四组比较患者生存。

**结果:**

142例患者，50例（35.2％）检出IKZF1基因缺失，其中4～7号外显子缺失占44.0％；与非缺失型比较，IKZF1缺失组B-ALL患者初诊时白细胞计数升高比例更高（52.0％对28.3％，*P*＝0.005）、诱导治疗第14天微小残留病转阴率更低（MRD_1_，40.0％对70.7％，*P*<0.001）以及Ph染色体阳性比例更高（52.0％对21.7％，*P*<0.001）。单因素分析显示，IKZF1缺失组3年EFS率、OS率明显低于IKZF1非缺失组［（37.1±7.3）％对（54.7±5.4）％，*P*＝0.025；（51.8±7.9）％对（73.9±4.7）％，*P*＝0.013］；多因素分析显示IKZF1缺失是影响B-ALL患者EFS（*HR*＝1.744，95％*CI* 1.082～2.812，*P*＝0.022）、OS（*HR*＝2.036，95％*CI* 1.119～3.705，*P*＝0.020）的独立危险因素。其中，A组的3年EFS、OS、无病生存率（DFS）均明显低于其他亚组。在IKZF1缺失患者中，接受异基因造血干细胞移植（allo-HSCT）组3年OS率为（67.9±10.4）％，3年EFS率为（46.6±10.5）％，均明显高于非移植组的（31.9±11.0）％和（26.7±9.7）％（*P*值分别为0.005、0.026）。

**结论:**

IKZF1基因缺失的B-ALL患者整体预后差，儿童样化疗方案不能完全纠正IZKF1缺失对预后的不良影响；儿童样方案联合allo-HSCT可显著改善IKZF1缺失B-ALL的整体预后。

## Abstract (en)

**Objective:**

This study aimed to investigate the prognostic significance of IKZF1 gene deletion in patients with acute B lymphoblastic leukemia（B-ALL）.

**Methods:**

The clinical data of 142 patients with B-ALL diagnosed in Nanfang Hospital between March 2016 and September 2019 were analyzed.

**Results:**

IKZF1 deletion was found in 36.0％ of the 142 patients with B-ALL, whereas exon 4–7 deletion was found in 44.0％. White blood cell counts were higher in patients with the IKZF1 deletion（52.0％ and 28.3％, *P*＝0.005）; these patients also experienced worse effects of mid-term induction therapy（40.0％ and 70.7％, *P*<0.001）and had a higher proportion of Philadelphia chromosome-positive（52.0％ and 21.7％, respectively, *P*<0.001）. Univariate analysis revealed that the 3-year overall survival rate（OS）and event-free survival rate（EFS）in the IKZF1 deletion group were significantly lower than the IKZF1 wild-type group［（37.1±7.3）％ *vs*（54.7±5.4）％,（51.8±7.9）％ *vs*（73.9±4.7）％; *P*＝0.025, 0.013, respectively］. Multivariable analysis showed that harboring IKZF1 deletion was an adverse factor of EFS and OS（*HR*＝1.744, 2.036; *P*＝0.022, 0.020, respectively）. Furthermore, the IKZF1 deletion/chemotherapy group had significantly lower 3-year OS, EFS, and disease-free survival rates than other subgroups. In the IKZF1 deletion cohort, allo-hematopoietic stem cell transplantation（HSCT）significantly improved OS and EFS compared to non-allo-HSCT［（67.9±10.4）％ *vs*（31.9±11.0）％,（46.6±10.5）％ *vs*（26.7±9.7）％; *P*＝0.005, 0.026, respectively］.

**Conclusion:**

Pediatric-inspired chemotherapy was unable to completely reverse the negative effect of IKZF1 deletion on prognosis. Pediatric-inspired regimen therapy combined with allo-HSCT, in contrast, significantly improved the overall prognosis of IKZF1 deletion B-ALL.

IKZF1基因编码锌指转录因子IKAROS，是最早被识别的淋系转录因子之一，负责调控造血干细胞向成熟淋巴细胞分化[1]–[2]。IKAROS蛋白含有6个锌指结构，其中N端的4个锌指结构位于维持肿瘤抑制和转录调控所必需的关键DNA结合域[3]–[4]；研究发现，编码上述关键锌指结构的IKZF1基因的大片段缺失或突变与急性淋巴细胞白血病（ALL）不良的预后密切相关。在儿童ALL中，IKZF1基因缺失发生率约为15％，在成人ALL中为30％～40％，在70％以上的Ph染色体阳性ALL中检测到IKZF1基因缺失或序列突变[5]–[8]。尽管IKZF1基因缺失或突变已作为预后分层的重要指标，但由于缺乏特异性靶向药物和治疗方法，目前基于IKZF1缺失/突变指导的治疗策略尚不清楚。本研究中，我们分析了本中心142例B-ALL患者的IKZF1基因缺失情况及其临床特征，并重点分析了异基因造血干细胞移植（allo-HSCT）对该类型患者生存的影响，探索基于IKZF1基因状态指导的治疗方案。

## 病例与方法

1. 研究对象：对2016年3月至2019年9月就诊于我院血液科的142例初诊B-ALL患者的临床资料进行回顾性分析。所有患者均经细胞形态学、免疫学、细胞遗传学和分子生物学（MICM）检测，诊断分型采用WHO2016年关于急性白血病的诊断标准。白血病细胞免疫分型及微小残留病（MRD）采用流式细胞术（FCM）检测，诱导化疗第14、24及巩固治疗前检测3次MRD水平，分别计为MRD_1_、MRD_2_、MRD_3_。将患者按IKZF1状态和治疗情况分为四组：IKZF1缺失/单纯化疗组（A组）、IKZF1缺失/移植组（B组）、IKZF1非缺失/单纯化疗（C组）、IKZF1非缺失/移植（D组），比较分析患者的生存情况。本研究经南方医科大学南方医院伦理审查委员会批准（批件号：NFEC-2018-002），并取得所有参与者的知情同意。

2. 跨越断裂点PCR检测IKZFl基因外显子缺失：参考Caye等[9]的研究，分别在IKZF1基因第2、4、7、8号外显子上设计基因组上、下游引物。Δ2-7及Δ2-8于第2号外显子上均存在缺失，但断裂位点不一致，故上述两种突变的上游引物需单独设计。突变上游引物命名为F.2a（5′-CAACAAGTGACCCATCCTTTG-3′）、F.2b（5′-CTCCTCTAATCTTTGGACTTGTGA-3′）、F.4（5′-GGAGTCTGTGAAGGTCACACC-3′），下游引物分别命名为R-7（5′-AAAGAACCCTCAGGCATTCA-3′）、R-8（5′-GTCTCGGCATACAGGGAAGA-3′）。引物及其合成产物的正确性，均在美国国立生物技术信息中心进行blast验证。引物以PAGE纯化，5′端加入FAM荧光基团修饰，3′端加入TAMRA荧光基团修饰，吸光度（*A*）值为2。按QIAamp DNA Micro Kit说明书提取基因组DNA。扩增反应参照ABI 7500型定量PCR仪使用说明书，设置反应程序，并收集荧光信号，自动设置Ct值，软件中相应出现标准扩增曲线且Ct值<36定义为阳性结果。

3. 治疗方案：所有ALL患者接受强化培门冬酶的儿童样方案PDT-ALL-2016[10]。诱导前接受地塞米松进行预治疗，接着予VICLP（长春新碱、去甲氧柔红霉素、培门冬酶、泼尼松）方案进行诱导缓解和CAM（环磷酰胺、阿糖胞苷和6-巯基嘌呤）模块治疗；明确Ph染色体阳性者诱导治疗第14天起加用酪氨酸激酶抑制剂治疗（达沙替尼 100 mg 每日1次），CD20阳性（流式细胞术免疫分型CD20阳性细胞比例>20％）患者加用利妥昔单抗（诱导期：375 mg/m^2^，每周1次，巩固期：375 mg/m^2^，每周2次）。诱导缓解后予阿糖胞苷、甲氨蝶呤、环磷酰胺联合培门冬酶巩固化疗模块1～3进行巩固治疗；综合患者诊断时IKZF1基因缺失等危险因素和基于不同时间点MRD评价的治疗反应进行危险分层，高危组患者进入allo-HSCT路径，结合患者意愿及供者情况决定是否选择allo-HSCT。移植预处理方案和GVHD防治根据本单位既定方案进行[11]。

4. 随访：采用查阅患者住院病历、门诊定期随访和电话随访，随访截止日期为2020年10月30日。无事件生存（EFS）期定义为自诊断到第1次事件（包括持续不缓解、复发、完全缓解期间因其他原因死亡）的时间。总生存（OS）期定义为自确诊至患者死亡或随访截止的时间。无病生存（DFS）期定义为完全缓解至复发、随访截止或患者死亡的时间。

5. 统计学处理：数据使用 SPSS 22.0 统计学软件进行分析，正态分布计量资料采用*x*±*s*表示，计数资料用例数、百分数表示，临床特征分析及比较使用卡方检验及方差分析，生存分析使用Kaplan-Meier生存曲线及Log-rank检验，多因素分析使用Cox回归模型。*P*<0.05为差异有统计学意义。

## 结果

1. 伴IKZF1基因缺失B-ALL患者临床及实验室特征：IKZF1基因缺失患者的临床资料见表1，50例（35.2％）患者检出IKZF1基因缺失，其中22例检测到4～7号外显子缺失，10例检测到2～7号外显子缺失，3例检测到2～8号外显子缺失，2例检测到4～8外显子缺失，13例为混合外显子缺失。与非缺失患者相比，IKZF1缺失组患者初诊时WBC≥30×10^9^/L患者比例更高（52.0％对28.3％，*P*＝0.005）、早期诱导MRD转阴率更低（MRD_1_，40.0％对70.7％，*P*<0.001）以及Ph染色体阳性比例更高（52.0％对21.7％，*P*<0.001）。

**表1:** IKZF1缺失急性B淋巴细胞白血病患者临床及实验室特征

特征	非IKZF1缺失（92例）	IKZF1缺失（50例）	检验值	*P*值
年龄［岁，*M*（范围）］	31（16～59）	32（17～51）	0.268^a^	0.605
性别（例，男/女）	47/45	26/24	0.011	0.917
WBC≥30×10^9^/L［例（％）］	26（28.3）	26（52.0）	7.866	0.005
LDH≥600 IU/L［例（％）］	35（38.0）	20（40.0）	0.052	0.858
CD20阳性［例（％）］	29（31.5）	18（36.0）	0.293	0.588
MRD_1_>1％［例（％）］	27（29.3）	30（60.0）	12.667	<0.001
MRD_2_>0.1％［例（％）］	21（22.8）	18（36.0）	2.822	0.093
MRD_3_>0.01％［例（％）］	20（21.7）	17（34.0）	3.035	0.081
首疗程完全缓解［例（％）］	74（80.4）	35（70.0）	1.977	0.160
Ph染色体阳性［例（％）］	20（21.7）	26（52.0）	13.545	<0.001

注：检验值中^a^为*F*值，其他均为*χ*^2^值；MRD_1_：化疗第14天微小残留病（MRD）水平；MRD_2_：化疗第24天MRD水平；MRD_3_：巩固治疗前MRD水平

2. 按IKZF1状态和治疗情况分组的四组患者基线临床资料分析：142例患者中，男73例（51.4％），女69例（48.6％），中位年龄31（18～64）岁。四组在性别、年龄、初诊时WBC、血清 LDH水平、髓外浸润、CD20表达、诱导期MRD水平、首疗程缓解情况、合并Ph染色体阳性等基线情况比较见表2。四组比较，初发WBC、诱导早期MRD阳性（MRD_1_>1％）、伴Ph染色体阳性、复发和死亡患者比例的差异均有统计学意义（*P*值均<0.05）。

**表2:** 四组急性B淋巴细胞白血病患者临床特征基线值比较

特征	IKZF1缺失组		IKZF1非缺失组		检验值	*P*值
	单纯化疗（24例）	allo-HSCT（26例）	单纯化疗（47例）	allo-HSCT（45例）		
年龄（岁，*x*±*s*）	32.3±8.3	29.7±6.3	33.2±8.7	30.1±7.9	1.654^a^	0.180
性别（例，男/女）	10/14	16/10	20/27	27/18	4.785	0.188
初诊WBC≥30×10^9^/L［例（％）］	14（58.3）	12（46.1）	14（29.8）	12（26.7）	8.760	0.033
初诊LDH≥600 IU/L［例（％）］	11（45.8）	9（34.6）	18（38.2）	17（37.8）	0.717	0.869
CD20阳性［例（％）］	9（37.5）	9（34.6）	14（29.8）	15（33.3）	0.471	0.925
MRD_1_>1％［例（％）］	12（50.0）	18（69.2）	13（27.7）	14（31.1）	14.702	0.002
MRD_2_>0.1％［例（％）］	7（29.2）	11（42.3）	9（19.1）	12（26.7）	4.556	0.207
MRD_3_>0.01％［例（％）］	7（29.2）	10（38.5）	11（23.4）	9（20.0）	3.225	0.358
首疗程完全缓解［例（％）］	18（75.0）	17（65.4）	38（80.9）	36（80.0）	2.633	0.452
Ph染色体阳性［例（％）］	12（50.0）	14（53.8）	11（23.4）	9（20.0）	13.751	0.003
复发［例（％）］	16（66.7）	8（30.7）	16（34.0）	14（31.1）	10.164	0.017
死亡［例（％）］	14（58.3）	7（26.9）	14（29.8）	10（22.2）	10.085	0.018

注：检验值中^a^为*F*值，其他均为*χ*^2^值。allo-HSCT：异基因造血干细胞移植；MRD_1_：化疗第14天微小残留病（MRD）水平；MRD_2_：化疗第24天MRD水平；MRD_3_：巩固治疗前MRD水平

3. 生存分析：四组患者生存比较结果见图1，A组3年EFS、OS、DFS率均明显低于其他三组，累积复发率（CIR）明显高于其他三组。IKZF1缺失患者中移植组的3年OS率及EFS率分别为（67.9±10.4）％、（46.6±10.5）％，非移植组的3年OS率及EFS率分别为（31.9±11.0）％、（26.7±9.7）％，差异均有统计学意义（*P*值分别为0.005、0.026）。

**图1:**
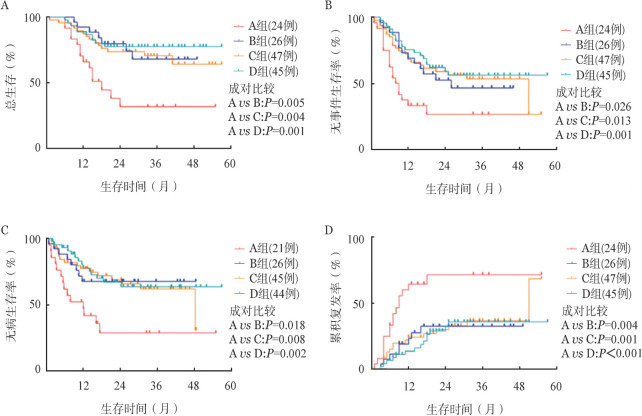
四组急性B淋巴细胞白血病患者总生存（A）、无事件生存（B）、无病生存（C）、累积复发（D）曲线比较 A组：IKZF1缺失/单纯化疗组；B组：IKZF1缺失/移植组；C组：IKZF1非缺失/单纯化疗组；D组：IKZF1非缺失/移植组

4. 影响B-ALL患者预后的单因素和多因素分析：对可能影响患者预后的相关因素进行单因素分析，发现初诊WBC、IKZF1缺失、Ph染色体阳性与预后相关（表3）。将单因素分析中*P*<0.05的因素纳入Cox回归模型进行多因素分析（表4），发现IKZF1缺失、CD20阳性是影响患者EFS的独立危险因素。年龄>35岁、IKZF1缺失是影响患者OS的独立危险因素，移植是OS的独立保护因素。

**表3:** 142例急性B淋巴细胞白血病患者预后单因素分析（％，*x*±*s*）

因素	例数	EFS		OS	
		3年EFS率	*P*值	3年OS率	*P*值
年龄			0.112		0.007
<35岁	92	54.7±5.4		75.2±4.6	
≥35岁	50	36.6±7.3		50.2±7.9	
性别			0.101		0.211
男	73	54.5±6.1		69.8±5.8	
女	69	42.1±6.3		63.0±6.1	
初诊WBC（×10^9^/L）			0.007		0.077
<30	90	56.4±5.5		73.7±4.7	
≥30	52	35.4±6.8		53.9±7.8	
血清LDH（IU/L）			0.758		0.985
<600	87	46.0±5.6		67.2±5.2	
≥600	55	53.3±6.9		65.5±7.1	
IKZF1基因			0.025		0.013
缺失	50	37.1±7.3		51.8±7.9	
非缺失	90	54.7±5.4		73.9±4.7	
CD20表达			0.022		0.449
阴性	95	55.5±5.4		69.1±5.1	
阳性	47	34.7±7.2		61.3±7.5	
Ph染色体			0.041		0.044
阴性	96	33.4±7.5		74.6±4.5	
阳性	46	55.5±5.3		50.7±8.5	
MRD_1_水平			0.211		0.615
≤1％	85	53.2±5.1		68.5±5.3	
>1％	57	41.7±6.8		63.8±6.8	
MRD_2_水平			0.231		0.400
≤0.1％	103	50.9±5.2		67.8±5.0	
>0.1％	39	42.7±8.1		63.3±7.8	
MRD_3_水平			0.491		0.615
≤0.01％	105	50.0±5.1		68.5±5.3	
>0.01％	37	44.3±8.4		63.8±6.8	
首疗程完全缓解			0.537		0.989
是	109	49.9±5.0		65.8±4.9	
否	33	44.4±8.8		68.9±8.2	
移植			0.127		0.038
是	71	44.6±6.1		58.9±6.2	
否	71	52.7±6.2		74.2±5.5	

注：EFS：无事件生存；OS：总生存；MRD_1_：化疗第14天微小残留病（MRD）水平；MRD_2_：化疗第24天MRD水平；MRD_3_：巩固治疗前MRD水平

**表4:** 142例急性B淋巴细胞白血病患者预后多因素分析

因素	EFS		OS	
	*HR*（95％*CI*）	*P*值	*HR*（95％*CI*）	*P*值
年龄>35岁	1.365（0.837～2.226）	0.212	1.875（1.031～3.409）	0.039
WBC≥30×10^9^/L	1.423（0.856～2.366）	0.174	1.268（0.681～2.361）	0.453
IKZF1基因缺失	1.744（1.082～2.812）	0.022	2.036（1.119～3.705）	0.020
CD20阳性	1.725（1.074～2.770）	0.024	1.325（0.718～2.445）	0.368
MRD_1_>1％	1.200（0.589～2.443）	0.615	0.971（0.380～2.485）	0.951
MRD_2_>0.1％	1.353（0.810～2.259）	0.248	1.403（0.734～2.683）	0.305
MRD_3_>0.01％	1.718（0.642～4.596）	0.281	1.152（0.428～3.102）	0.780
伴Ph染色体阳性	1.127（0.625～2.031）	0.692	1.070（0.505～2.268）	0.850
移植	0.637（0.397～1.023）	0.062	0.515（0.270～0.981）	0.044

注：EFS：无事件生存；OS：总生存；MRD_1_：化疗第14天微小残留病（MRD）水平；MRD_2_：化疗第24天MRD水平；MRD_3_：巩固治疗前MRD水平

## 讨论

IKZF1基因位于染色体7p12.2，编码IKAROS蛋白，是造血过程中的重要转录因子，主要调控T和B淋巴细胞的正常发育分化。IKZF1基因缺失在高危B-ALL发病机制中扮演重要角色，参与B细胞分化阻滞、代谢重编程、白血病微环境黏附、疾病的复发和耐药等过程。由于目前缺乏特异性靶向药物，探索IKZF1缺失B-ALL的治疗策略有重要意义[12]–[14]。

近年来，随着研究深入，IKZF1基因缺失对B-ALL的诱导缓解、疾病复发和长期生存等重要影响逐渐被阐明。美国St Jude儿童医院Mullighan团队[7]采用高通量基因组测序分析，分别在两个独立队列中进行检测和验证，证实IKZF1缺失或突变与B-ALL不良预后有重要关联；德国GMALL研究组[15]2017年进一步研究报道，外显子大片段缺失导致IKZF1基因功能丧失，IKZF1基因的突变负荷与预后密切相关，高突变负荷与预后不良相关；与国外研究结果相似，我们在本研究中对IKZF1缺失ALL患者的临床特征进行了分析，发现IKZF1缺失患者的早期诱导效果不佳，首次化疗完全缓解率低，生存分析显示IKZF1缺失组较非缺失组OS和EFS期明显缩短。

由于目前尚无特异性针对IKZF1缺失的靶向治疗药物，IKZF1缺失患者单纯化疗预后不佳[16]，包括免疫治疗都不能完全纠正IKZF1缺失的不良预后[17]，因此，探索联合策略改善IKZF1缺失高危B-ALL预后具有重要意义。目前多项研究报道了allo-HSCT可以改善IKZF1缺失ALL的预后。Dhédin等[18]报道了206例前体B细胞ALL患者的预后，allo-HSCT可以使IKZF1缺失患者获益（*HR*＝0.42）。苏州大学附属第一医院血液科分析了164例成人B-ALL患者，结果显示allo-HSCT可以改善IKZF1突变尤其是 Ph染色体阳性ALL的OS和LFS[19]；但单纯移植是否能完全克服IKZF1不良预后，目前尚不完全清楚。

本研究中我们采用了PDT-ALL-2016的儿童样化疗方案，相比IKZF1缺失组，非IKZF1缺失组不论是否接受allo-HSCT，整体生存达到了70％～80％，显示儿童样化疗方案PDT-ALL-2016使成人B-ALL患者获得了比较理想的生存；但在IKZF1缺失亚组，IKZF1缺失/单纯化疗组对比其他三组预后明显较差，表明仅使用儿童样化疗，不足以克服IKZF1缺失带来的不良影响。在IKZF1缺失患者中，非移植组的预后显著差于移植组，提示在现阶段的治疗方案下，allo-HSCT仍是可以使IKZF1缺失ALL患者获益的首要治疗手段。相比国内外已有报道，本研究针对IKZF1缺失高危B-ALL采用了儿童样化疗方案联合allo-HSCT的治疗策略，初步结果显示，联合策略有可能改善IKZF1基因缺失的高危B-ALL的不良预后。但本研究仅为单中心分析，对于allo-HSCT治疗后长期的不良事件及存活情况还需进行后续深入研究。

综上所述，IKZF1基因缺失B-ALL患者整体预后差，儿童样化疗方案联合allo-HSCT有望克服IKZF1基因缺失对预后的不良影响，该结论有待多中心数据的进一步验证；同时，针对IKZF1缺失分子机制的揭示和靶向药物的研发，将进一步改善IKZF1缺失B-ALL的临床预后。
